# Prevalence of submicroscopic congenital malaria and associated risk factors in an area of low endemicity of Guatemala

**DOI:** 10.1186/s12936-025-05670-6

**Published:** 2025-11-27

**Authors:** Rubi Gordillo-Franco, Lucía Ortiz, Azucena Bardají, David Castañeda, Clara Menendez, Norma Padilla, María Eugenia Castellanos

**Affiliations:** 1 Universidad del Valle de Guatemala, Guatemala City, Guatemala; 2 ISGlobal, Barcelona, Spain; 3 Facultat de Medicina I Ciències de La Salut, Universitat de Barcelona (UB), Barcelona, Spain; 4 Centro de Investigação Em Saúde de Manhiça (CISM), Maputo, Mozambique; 5 Centro de Investigación Biomédica en Red de Epidemiología y Salud Pública, Instituto de Salud Carlos III, Madrid, Spain; 6 Public Health and Tropical Medicine, College of Medicine and Dentistry, James Cook University, Townsville, QLD Australia; 7 Present Address: Department of Microbiology and Immunology, School of Medicine, Emory University, 1510 Clifton Rd, Atlanta, GA 30322 USA; 8 Present Address: Universidad Rafael Landívar, Guatemala City, Guatemala

**Keywords:** Congenital malaria, Submicroscopic, Epidemiology, Risk factors

## Abstract

**Background:**

Malaria during pregnancy can have severe consequences, such as abortion, intrauterine growth restriction, low birth weight, maternal and fetal anaemia, congenital malaria and fetal death. Submicroscopic congenital malaria is traditionally defined as a *Plasmodium* infection detected only by molecular methods in the umbilical cord blood and/or in the newborn peripheral blood. Data on the prevalence and risk factors associated with submicroscopic malaria in pregnant women from Latin America are scarce. This study aimed to describe the first submicroscopic infection rate by *Plasmodium* sp. in umbilical cord blood in an area of low malaria endemicity and the risk factors that are associated with congenital malaria transmission.

**Methods:**

This study used samples and data from a prospective cohort study of pregnant women in Fray Bartolomé de las Casas, Guatemala, between 2009 and 2011. A random sample of umbilical cord samples (negative by microscopy) was tested using quantitative real-time polymerase chain reaction (qPCR) to determine the prevalence of submicroscopic *Plasmodium* sp. infections. A case–control study was then conducted to identify the risk factors in this population associated with submicroscopic congenital malaria. The cases were newborns with an umbilical cord sample confirmed with submicroscopic malaria infection. The controls consisted of newborns and umbilical cord samples, which yielded negative results for malaria infection by both microscopy and qPCR.

**Results:**

A total of 442 cord samples from a subsample of 480 randomly selected samples were analysed by qPCR (92%). Among these samples, 54 tested positive for malaria, resulting in a submicroscopic cord blood malaria prevalence of 12.2% (95% CI 9.3% -15.6%). The case–control study included 54 cases and 51 controls. Submicroscopic maternal *Plasmodium* sp. infection (aOR 7.55, 95% CI 1.84–51.5) and gravidity (aOR 1.49, 95% CI 1.13–2.04) were associated with a higher risk of submicroscopic congenital malaria infection.

**Conclusion:**

Submicroscopic malaria infection in pregnancy was shown to be associated with risk of submicroscopic congenital malaria infection. Given the deleterious effects observed of any malaria infection in pregnancy, prompt diagnosis and treatment of malaria infection should be emphasized.

**Supplementary Information:**

The online version contains supplementary material available at https://doi.org/10.1186/s12936-025-05670-6.

## Background

Despite the reduction in the prevalence of malaria, it continues to be a major cause of morbidity and mortality worldwide. Malaria diagnosis is challenging; studies that have compared the presence of *Plasmodium* detected by microscopy and molecular methods have found significant differences [1–5]. Results suggest that PCR can detect 50% or more cases than microscopy [1, 2]. The proportion of people infected with *Plasmodium* but detectable only by molecular methods is described as having submicroscopic infection or submicroscopic malaria [1, 5–9]. These submicroscopic infections are more likely to occur in areas that have experienced a significant decline in malaria transmission over the past decade, rather than in historically high-endemic regions where repeated exposure often leads to acquired immunity [1, 8–14].

In the past decades, submicroscopic infection has been described during pregnancy in both the mother and the newborn [3–6, 15–17]. Reported prevalences of *Plasmodium* sp. submicroscopic malaria during pregnancy in empirical studies have ranged from 4.6% to 70.8% in Guatemala, Colombia, the Democratic Republic of the Congo, and on the Thailand–Myanmar border [3, 8, 16, 18, 19]. Recently, a large meta-analysis estimated a median overall prevalence of 13.5% for submicroscopic malaria in pregnancy [5]. By region, the highest value was observed in Africa (median 18.6%) and the lowest in the Americas (6.0%). Importantly, in the Americas, the risk of submicroscopic malaria in pregnancy was higher for *Plasmodium vivax* than for *Plasmodium falciparum* infection [5].

Both peripheral and placental *Plasmodium* submicroscopic infections, particularly those by *P. falciparum*, have been associated with pregnancy and fetal outcomes, such as maternal anaemia, premature delivery, low birth weight, small for gestational age and congenital malaria [6, 20–22]. Congenital malaria in malaria-endemic settings has been defined as the presence of malaria parasites in the peripheral blood of the newborn or in the cord blood smear at delivery [23, 24]. A meta-analysis described a global prevalence of congenital malaria of 6.9%, but it ranged from 0% in Colombia to 46.7% in Nigeria [24]. Also, these variabilities might be related to the type of sample tested (cord blood, newborn peripheral blood or both), transmission settings of malaria (stable or unstable) in the study area and the diagnostic method used (microscopy, rapid diagnostic test, or PCR) [24–27]. For instance, a meta-analysis conducted on studies from Colombia found a prevalence of 1.3% for congenital malaria using thick blood smears and 16.2% using PCR [4].

Considering that 19% of pregnancies in countries with unstable malaria transmission in Latin America and the Caribbean are at risk of *P. vivax* infection, malaria in pregnancy and congenital malaria continue to be critical public health issues that warrants further attention in the Americas [28]. In Guatemala, these estimates indicate that the country had approximately 300,000 at-risk pregnancies for *P. vivax* in areas of unstable transmission in 2020 [28].

The study “*Plasmodium vivax* Infection in Pregnancy (*PregVax*)” not only documented the first case of congenital malaria by microscopy in Guatemala but also discovered that among 900 cord blood samples, only 1 (0.1%, n = 1/900) was positive by microscopy for *Plasmodium* sp, whereas 14.8% (n = 15/101) were positive by PCR [3, 29]. Still, scarce information exists on the burden of submicroscopic malaria during pregnancy in *P. vivax*-endemic settings such as Guatemala and other regions of Latin America. Guatemala continues to screen pregnant women with microscopy but does not routinely test newborns (peripheral blood or umbilical cord). As a result, the actual burden of submicroscopic congenital malaria could be underestimated. As the PCR results in the *PregVax* study were based on a small sample (n = 101), the present study aimed to expand these findings by providing a more comprehensive estimate of the submicroscopic infection rate by *Plasmodium* sp. in umbilical cord blood samples and to determine the risk factors associated with submicroscopic congenital malaria transmission in Guatemala. Findings from this research will provide critical evidence to inform screening protocols and prevention strategies in antenatal care throughout the region, thereby enhancing efforts to eliminate transmission by addressing previously overlooked pathways.

## Methods

### Study design

This study used samples and data from *PregVax,* a prospective cohort study of pregnant women in Fray Bartolomé de las Casas, Guatemala, between 2009 and 2011 [3]. Using a cross-sectional design, a random sample of umbilical cord samples (all negative by microscopy) was tested using qPCR to establish the prevalence of submicroscopic *Plasmodium* sp. infections. A case–control study was then conducted to determine risk factors associated with submicroscopic congenital malaria. The current study was initiated in 2015, several years after the primary research concluded, so no antimalarial treatment was provided to women or children with a positive malaria molecular result.

### Study site

Guatemala is located in Central America, with a territorial area of 108,889 km^2^. The territory is divided into 22 departments, which in turn are divided into 340 municipalities [30]. The study was conducted in the municipality of Fray Bartolomé de Las Casas, a rural, low-transmission malaria-endemic area in the department of Alta Verapaz, which has a land area of 8686 km^2^ [30]. The climate in the municipality is hot and humid, with two distinct seasons: a dry season from March to May and a rainy season for the remainder of the year [31]. The incidence of malaria in Guatemala has decreased by almost 91% since 2000, only 7,384 malaria cases were reported during 2010 [32]. The most common species are *P. vivax* and *P. falciparum,* with *P. vivax* being responsible for 98% of the cases [33].

### Description of primary study

*‘Plasmodium vivax* Infection in Pregnancy (*PregVax*)’ was a multicentre study in Brazil, Colombia, Guatemala, India and Papua Guinea, which began in March 2008 and ended in August 2012. The methodology and main findings of the study have been previously published [3]. Briefly, at the time of participant recruitment, a standardised questionnaire was filled out to collect demographic, obstetric, and clinical information from pregnant women attending routine antenatal care at the Fray Bartolomé de las Casas (FBC) District Hospital. At each follow-up visit (maximum 3) and at the time of delivery, data on symptoms suggestive of malaria was collected and a blood sample was drawn to prepare a thin and thick blood smears and two blood samples on filter paper to determine the presence of *Plasmodium* sp. by molecular methods; haemoglobin (Hb) levels were measured in the same sample. Blood was also collected from the placenta and umbilical cord at delivery, and the same samples were prepared (thin and thick blood smears and filter paper). A blood sample was drawn from the newborn within the first 24–48 h of life for thin and thick blood smears, as well as two filter papers, to determine parasitaemia and identify *Plasmodium* sp. The thick blood smear slides were stained with Giemsa, and each slide was evaluated by two independent microscopists using light microscopy. A third microscopist resolved any discrepancies. Parasitic density was reported as the number of asexual or sexual forms of *Plasmodium*/μl of blood. All women with positive microscopy results were treated according to national guidelines. The filter papers were kept to further identify *Plasmodium* through molecular methods.

### Sampling for this study

***Cross-sectional study***. Of the 2009 women recruited in the *PregVax* cohort study, only 831 women had a complete set of samples collected during recruitment and delivery (mother’s peripheral blood, placental blood, umbilical cord blood, newborn’s peripheral blood). At the Universidad del Valle de Guatemala facilities, 480 cord blood samples from these sets were randomly selected using OpenEpi, a software that generates random numbers [34]. The sample size was calculated using the standard formula for estimating a population proportion. A hypothesized prevalence of submicroscopic malaria infection in the population of 50% (the most conservative number) was assumed, resulting in a sample size of 385. A ~ 25% adjustment was made to account for blood filter papers with insufficient DNA yield for molecular testing, increasing the sample size to 480 cord blood samples. None of these samples was *Plasmodium*-positive by microscopy, so they were used to determine the prevalence of submicroscopic malaria in umbilical cord blood.

***Case–control study***. Of the sets with randomly selected umbilical cord samples, all those with a qPCR-positive umbilical cord were included in the case–control study to identify risk factors associated with submicroscopic congenital malaria. The cases were newborns with an umbilical cord sample confirmed with submicroscopic malaria infection (negative malaria results by microscopy and positive malaria results by qPCR). The controls consisted of newborns with negative malaria results, as determined by both microscopy and qPCR, in both umbilical cord and peripheral blood samples. Each control was selected preferentially according to the location of the case.

### Laboratory procedures

For this study, dried blood spots on filter paper from the following samples were analysed by qPCR for submicroscopic malaria: (a) randomly selected cord blood samples and (b) the corresponding mother’s peripheral blood, placental blood, newborn’s peripheral blood of the participants in which a qPCR-positive malaria cord blood sample was obtained. A genus-specific qPCR was performed on all samples [35, 36]. In those with a positive *Plasmodium* sp. result, a species-specific qPCR was carried out to detect *P. vivax* and *P. falciparum* [37]. The microscopy data obtained in the primary study confirmed the negative malaria result by microscopy in the analysed samples.

### Analytical strategy

The demographic and clinical characteristics of the participants were summarised using counts and percentages (categorical data) or measures of central tendency and dispersion (numerical data). The prevalence of submicroscopic malaria in the umbilical cord was calculated using the number of positive umbilical cords for *Plasmodium* sp. by qPCR as the numerator and the total number of analysed umbilical cords as the denominator.

Risk factors for submicroscopic congenital malaria infection were identified through logistic regression analysis. Potential risk factors considered were maternal diagnosis of submicroscopic malaria (by qPCR) during pregnancy, number of prenatal visits, gestational age at the time of recruitment, use of a bed net with or without insecticide, presence of malaria symptoms and anaemia. Anaemia was defined using World Health Organization (WHO) criteria, which classifies anaemia as follows: mild (10–10.9 mg/dL), moderate (7–9.9 mg/dL) and severe (less than 7 mg/dL) [38]. Age and number of pregnancies were considered possible confounding factors in the analysis and included in all multivariate models.

Crude odds ratios (ORs) with 95% confidence intervals (CIs) were estimated in the univariate analysis to evaluate the effect of each independent variable on the outcome variable and to determine which variables to include in the multivariate logistic regression analysis. To obtain the adjusted odds ratio (aOR), only independent variables with a *p*-value of 0.20 or less in the bi-variable logistic regression were included in the multivariable logistic regression.

Data from the *PregVax* questionnaire collected at the recruitment stage were merged with other *PregVax* databases (delivery, prenatal visits, and newborn) and with laboratory results to create a final database for this study. All analyses were performed using R version 3.6.2 [39]. The package “gtsummary” was used to create and report the descriptive results and summary tables [40].

## Results

### Cross-sectional study: prevalence of *Plasmodium* sp. sub-microscopic infections in umbilical cord blood

**Fig. 1:**
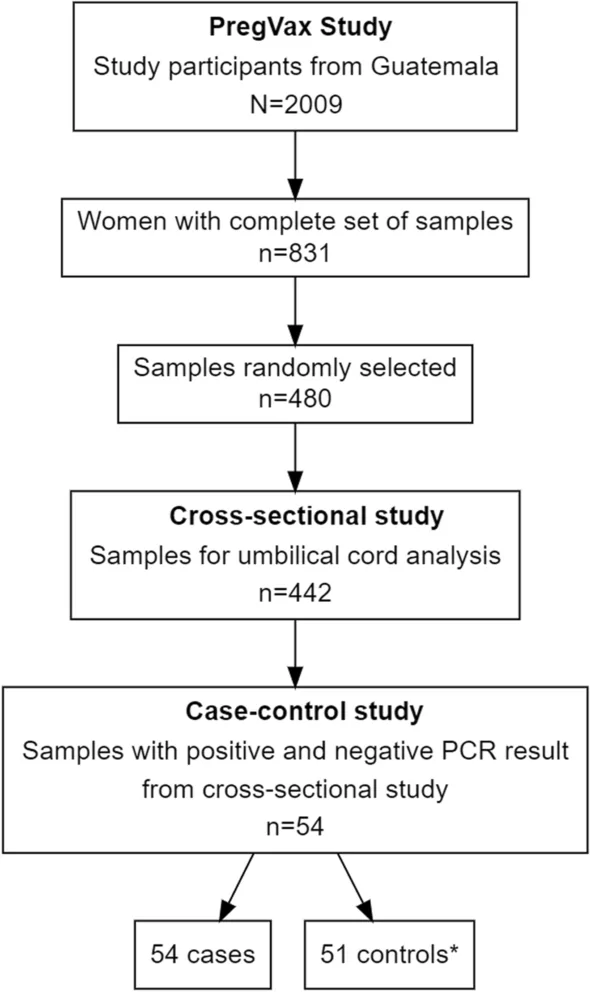
Process of selection of participants or samples from the *PregVax s*tudy for the cross-sectional and case–control study. *Due to time and resource constraints, only 49 paired samples were matched by location. For the remaining five cases, only two additional controls were included. Thus, a total of 54 cases and 51 controls were analyzed

qPCR laboratory testing was not performed on 38 umbilical cord samples due to human resources constraints; therefore, the final analysis included data from 442 participants (Fig. 1).

At recruitment, the median age of the pregnant women was 24 years (IQR 19, 29). Most women (79%) were recruited at their first or second prenatal visit and had a median gestational age of 27 weeks (IQR 20,33) (Table 1). The demographic and clinical characteristics of the 442 women included in the cross-sectional study were representative of those of all 2,009 study participants from Guatemala (Supplemental material, Table S1).

**Table 1:** Characteristics of the pregnant women during recruitment for the cross-sectional study, Guatemala, 2009–2011

Characteristic	N = 442
Age in years, median (IQR)	24 (19, 29)
Number of attended prenatal visits when enrolled, n, (%)	
1–2 visits	348 (79%)
> 2 visits	93 (21%)
Weight in pounds, (median, IQR)	126 (116, 143)
Height in centimetres (median, IQR)	151 (147, 155)
Haemoglobin at recruitment (g/dL) (median, IQR)	11.30 (10.70, 12.00)
Gravidity, (median, IQR)	2 (1, 4)
Gestational age in weeks at time of recruitment (median, IQR)	27 (20, 33)
Trimester of pregnancy at time of recruitment (according to gestational age), n (%)	
First	23 (5.2%)
Second	196 (45%)
Third	220 (50%)
Method to estimate gestational age, n (%)	
Date of last menstruation	9 (2.1%)
Ultrasound	78 (18%)
Uterine height	349 (80%)

± Observations with missing data: Number of prenatal visits when enrolled (n = 1), haemoglobin (n = 58), gravidity (n = 1), gestational age (n = 3), trimester of pregnancy according to gestational age (n = 3) and method to estimate gestational age (n = 6)

**Fig. 2:**
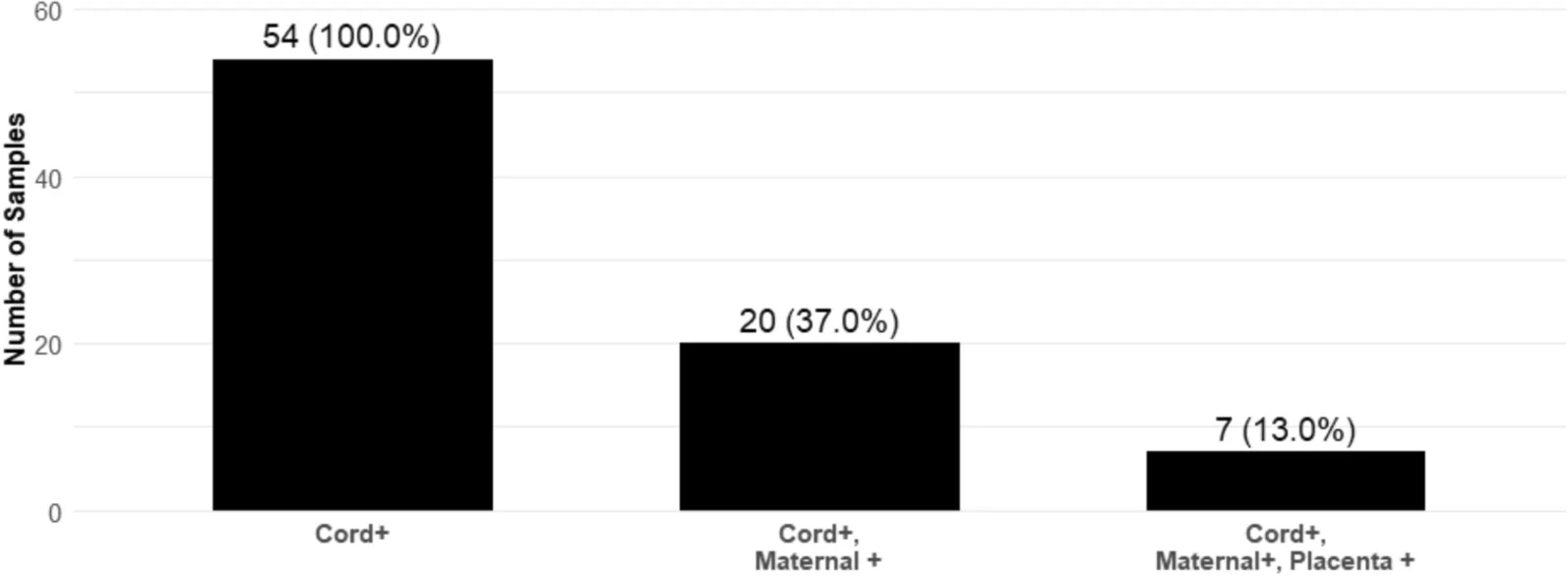
Frequency and proportion of study cases with positive qPCR results in other samples (maternal peripheral blood at enrolment and/or at delivery, placental blood)

**Fig. 3:**
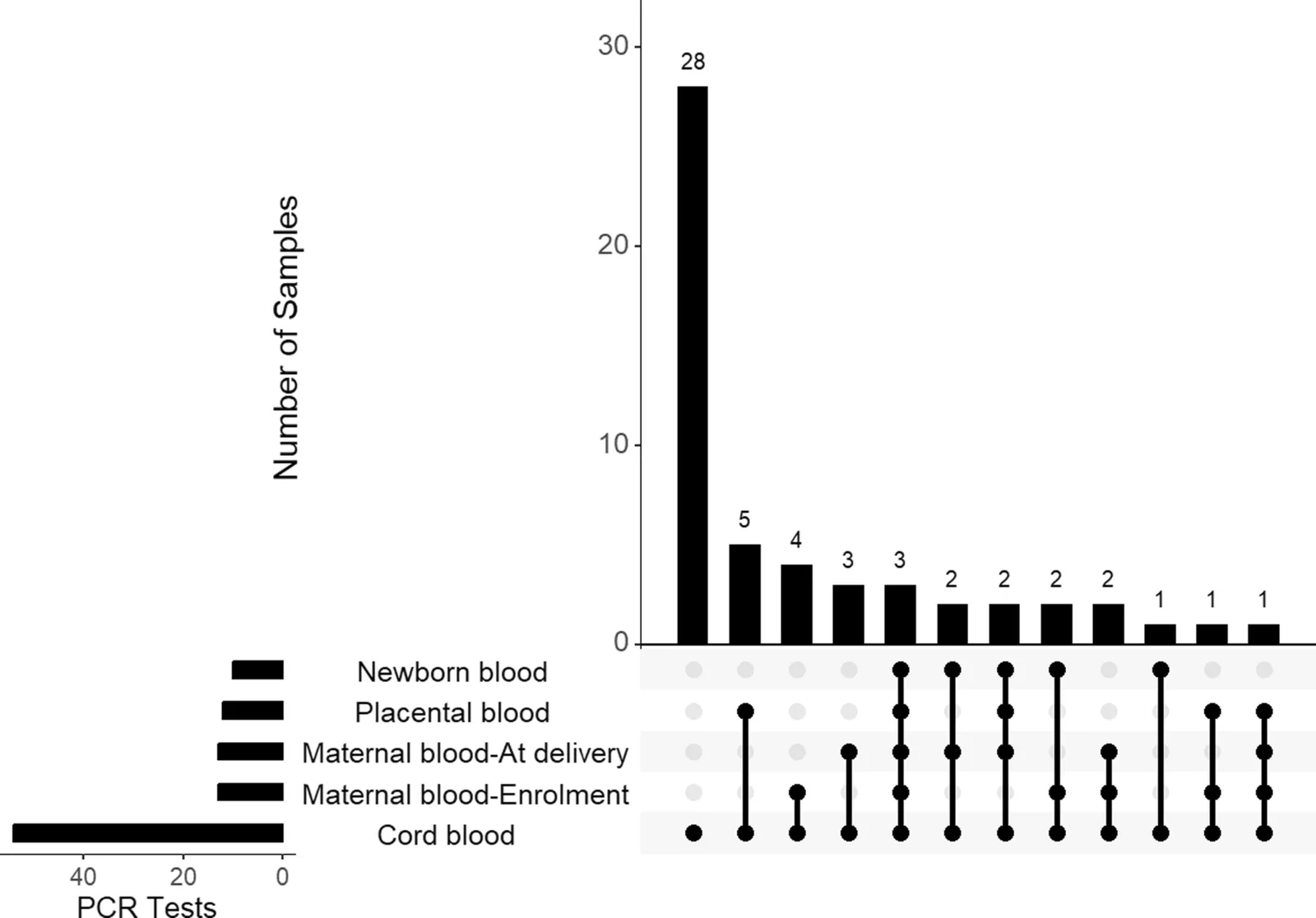
Distribution of qPCR-positive results among the 54 study cases. All cases, by definition, have a malaria-positive sample from the umbilical cord

Among the 442 umbilical cord samples, 54 tested positive for *Plasmodium* sp by qPCR, resulting in a submicroscopic cord blood malaria prevalence of 12.2% (95% CI 9.3% -15.6%). Also, 20 of the 54 cases (37%) had a positive peripheral maternal blood sample (at enrolment or delivery). Seven of these cases also had a positive result for the placental blood sample (Fig. 2). In three cases, all tested samples (peripheral blood from the newborn, maternal peripheral blood at recruitment and delivery, umbilical cord, and placental blood) were positive for malaria (Fig. 3). Figure 3 shows the distribution of qPCR-positive results for all the potential combinations among the 54 study cases.

The *Plasmodium* species could be identified only in 16 of these 54 cases. Thirteen (81%) cases had a mono-infection with *P. vivax*, one (6%) had a mono-infection with *P. falciparum*, and two (12%) had a *P.vivax/P. falciparum* coinfection.

### Case–control study: risk factors for congenital malaria

A total of 54 cases and 51 controls were enrolled, who met the study's case and control definitions (Fig. 1). Over half of the newborns were male (53% in controls, 54% in cases). Most cases and controls were born full term, only 4% (n = 2) of cases were born preterm (Table 2). The mean age of the mothers of the cases was 25 years, and of the controls 23 years; the weight, height, and haemoglobin at recruitment had similar values (Table 2). The median number of pregnancies for the mother of the cases was 3 (IQR 1.5), while for the mothers of the controls, it was 2 (IQR 1.3). The proportion of mothers with anaemia at delivery (35%) was higher in the cases than in the controls (22%), with a larger proportion of mothers of the cases with mild anaemia (25% vs 11%).

**Table 2:** Characteristics of pregnant women and newborns for the case–control study, Guatemala, 2009–2011

Characteristic	Controls, N = 51^a^	Cases, N = 54^a^
Maternal characteristics		
Age in years, median (IQR)	23 (20, 32)	25 (19, 31)
Number of prenatal visits when enrolled, n, (%)		
1–2 visits	42 (82%)	38 (72%)
> 2 visits	9 (18%)	15 (28%)
Gravidity, (median, IQR)	2 (1, 3)	3 (1, 5)
Gestational age in weeks at the time of recruitment, median (IQR)	28 (23, 35)	28 (24, 35)
Weight in pounds, median (IQR)	130 (118, 148)	128 (116, 144)
Height in centimetres, median (IQR)	150 (147, 155)	150 (148, 154)
Haemoglobin (g/dL) at recruitment, median (IQR)	11.30 (10.65, 11.95)	11.10 (10.30, 12.20)
Anaemia at delivery, n (%)		
No anaemia	35 (78%)	34 (65%)
Mild (10–10.9 haemoglobin g/dL)	5 (11%)	13 (25%)
Moderate (7–9.9 haemoglobin g/dL)	5 (11%)	5 (9.6%)
Newborn characteristics		
Sex, n (%)		
Male	27 (53%)	29 (54%)
Female	24 (47%)	25 (46%)
Temperature at birth in Celsius, median (IQR)	36.20 (36.00, 36.50)	36.20 (36.00, 36.38)
Length in centimetres, median (IQR)	50.00 (48.00, 51.00)	49.00 (48.00, 51.00)
Birthweight in grams, median (IQR)	3,100 (2,777, 3,419)	3,145 (2,813, 3,389)
Head circumference in centimetres, median (IQR)	34 (33, 35)	33 (32, 34)
Gestational age determined by the Ballard method		
30–35 weeks	0 (0%)	2 (4%)
36–40 weeks	27 (53%)	28 (52%)
> 40 weeks	24 (47%)	24 (44%)

^a^Cases: umbilical cord positive for *Plasmodium sp.* by qPCR, Controls: umbilical cord negative for *Plasmodium sp.* by qPCR

± Observations with missing data: Number of prenatal visits when enrolled (n = 1 for a case), gestational age at recruitment (n = 1 for a case), haemoglobin at recruitment (n = 8 for controls and n = 3 for cases) and anaemia at delivery (n = 6 for controls and n = 2 for cases)

In multivariate analysis, the risk of developing submicroscopic congenital malaria was 7.55 times higher in newborns whose mothers had a positive (by qPCR) maternal blood sample for malaria during pregnancy at recruitment (aOR 7.55, 95% CI 1.84–51.5) (Table 3). The gravidity of the mother also showed an association with submicroscopic congenital malaria (aOR 1.49, 95% CI 1.13–2.04). More cases than controls (24% vs 9.8%) had a positive (by qPCR) maternal blood sample for malaria at delivery; however, it cannot be concluded that there is an association based on the wide 95% CI (aOR = 2.47, 95% CI 0.73–9.40). Both cases and controls reported high net use and reported using nets treated with insecticide; these factors were not associated with submicroscopic congenital malaria (Table 3).

**Table 3:** Univariate and multivariate Analysis. Risk factors for submicroscopic congenital malaria, Guatemala, 2009–2011

	Univariate			Multivariate		
Characteristic	OR^a^	95% CI^b^	*p*-value	aOR^c^	95% CI^b^	*p*-value
Age, years	1.01	0.95, 1.07	0.717	0.91	0.82, 1.00	0.062
Gravidity	1.18	1.00, 1.41	0.057	1.49	1.13, 2.04	0.007
Gestational age in weeks at time of recruitment	1.00	0.96, 1.05	0.903	0.98	0.93, 1.03	0.448
Reported at enrolment to sleep under a bednet	1.45	0.30, 7.67	0.640			
Reported at enrolment that the bednet used to sleep on has insecticide	0.95	0.36, 2.50	0.922			
Peripheral maternal blood positive at recruitment	7.77	2.00, 51.5	0.009	7.55	1.84, 51.5	0.013
Peripheral maternal blood positive at delivery	2.92	1.01, 9.74	0.060	2.47	0.73, 9.40	0.158

^a^OR = Crude odds Ratio

^b^CI = Confidence Interval

^c^aOR = Adjusted Odds Ratio

## Discussion

This study described the prevalence of submicroscopic malaria infection in umbilical cord blood samples and determined risk factors associated with submicroscopic congenital malaria in Guatemala.

Submicroscopic malaria is of high interest in public health, as these infected individuals may become reservoirs contributing to the transmission of the parasite and may present with relapsing and chronic, low-density infections [1, 9, 41, 42].

Most research on congenital malaria comes from studies in Asia and Africa. The prevalence of 12% of submicroscopic congenital malaria, based on the detection of malaria parasites in the umbilical cord by qPCR, closely resembles that of a study conducted in Colombia, which found a prevalence, using PCR, of 12.2% in the umbilical cord and 16.2% in newborn peripheral blood [27]. Differences in the prevalence of congenital malaria between studies are expected, which may be due to various factors, including study design, study population, case definition of congenital malaria, seasonality, transmission settings, prevalent species and the method used to detect infection.

The published data on the long-term clinical impact of submicroscopic congenital malaria remains scarce. A previous study conducted in Burkina Faso did not find an association of submicroscopic *P. falciparum* congenital malaria with low birth weight, fever, and risk of malaria during the first two months of life [43]. In this study, 80% of the cases were *P. vivax* mono-infections, and there were no significant differences in neonatal outcomes between the control and case groups. Nonetheless, recent evidence from a large case-cohort study on the Thailand-Myanmar border suggests that submicroscopic *P. vivax* infection in pregnancy might be associated with lower infant birth weight (− 77 g, 95% CI − 163–10, *p* = 0.082) [18]. More studies are needed with longer follow-up time to investigate the potential long-term consequences of other clinical outcomes, such as cognitive development or behavioural functions [44].

Concerning risk factors for submicroscopic congenital malaria, the findings showed a strong association between maternal submicroscopic infection and the development of submicroscopic congenital malaria, particularly when the infection is present during pregnancy rather than at delivery. In this study, there was a 7.5-fold risk for submicroscopic congenital malaria in newborns of *Plasmodium*-positive women during pregnancy. These findings align with those reported in a Colombian study, which found a highly significant correlation (*p* < 0.001) between qPCR results in maternal peripheral blood samples and umbilical cord samples, as 59% of positive umbilical cords corresponded to positive maternal samples [45].

A positive association was observed between mother’s gravidity and submicroscopic congenital malaria. Pregnancy is a stage with a higher risk of malaria infection due to immunological and hormonal changes. In addition, *P. falciparum* can infect red blood cells and sequester them towards the developing placenta, causing placental inflammation and chronic infection [46, 47]. In the case of *P. vivax*, the preferred targets are reticulocytes that emerge from the spleen and the bone marrow [42, 48]. The spleen becomes a major reservoir for asexual parasites, contributing to recurrent and chronic infections, systemic inflammation and anaemia [42]. A study in Brazil found that infection with *P. vivax* was associated with placental lesions and an imbalance in homeostasis, as well as altered angiogenic factors, leptin, and cytokines [49]. These placental abnormalities have been linked to adverse outcomes in *P. falciparum*. Therefore, it could be hypothesized that when caused by *P. vivax,* they might be associated with the same adverse consequences, as shown by Carmona-Fonseca and Cardona Arias when comparing placental abnormalities between *P. vivax* and P. *falciparum* [50].

Susceptibility to malaria in pregnant women is higher in the first pregnancy, but the risk might remain in consecutive pregnancies [18, 51]. Studies confirm that antibodies directed against infected erythrocytes are usually absent during the first pregnancy, so there is a strong negative correlation between parity and susceptibility to infection by *P. falciparum;* however, immunity acquisition in *P. vivax* infections is still uncertain and inconsistently reported*.* [47, 49, 52]. Maternal age has previously been described as a risk factor for congenital malaria [53]. However, no significant associations were found in this investigation.

As more settings aim for malaria elimination, it may be necessary to revise or enhance existing health policies to strengthen programs for controlling and detecting malaria cases, including those involving submicroscopic or asymptomatic infections. People with these infections pose a significant obstacle to achieving elimination, as they continue to act as important reservoirs [41]. Also, it has been reported that pregnant women with submicroscopic infections have a higher risk of presenting subsequent microscopic infections [18].

Currently, implementing molecular screening in low-income settings might be challenging due to its high costs. Nevertheless, novel technologies such as ultrasensitive loop-mediated isothermal amplification assays might be considered in countries aiming for malaria elimination [54]. Additionally, in settings such as Guatemala, it is warranted to increase antenatal care among pregnant women, which was reported to be as low as 18% among poor women in Guatemala [55]. This increase in antenatal care also provides the opportunity to expand the scope of chemoprophylaxis, which for *P. vivax* is currently recommended to be weekly chloroquine in pregnant and breastfeeding women [56].

This study has several limitations. First, the data were collected over ten years ago. However, the data were collected from the only cohort study conducted in Guatemalan pregnant women to investigate the burden of malaria in pregnancy. Therefore, these data provide evidence that serves as a critical baseline for understanding the burden of submicroscopic infections and the associated risk factors in low-endemicity settings. Second, most pregnant women were enrolled during the second or third trimester with limited numbers of ANC visits, resulting in a lack of periodic malaria laboratory results. That information could have been used to determine infection rates and risk factors by trimester, and to gain insight into women with chronic maternal infection. Third, the estimation of the prevalence of submicroscopic congenital malaria relied exclusively on results from the umbilical cord. Diagnosing congenital malaria also includes detecting parasites at birth and up to the seventh day of life. Thus, these findings, which are limited to umbilical cord samples, may underestimate the prevalence of congenital malaria. Fourth, although all umbilical cords positive for submicroscopic infection in the case–control study were included, only one control was selected for each case, which might have reduced the statistical power of the study [57]. Finally, the *Plasmodium* species causing the malaria infection could not be fully characterized, as most cases were detected only at the genus level. Submicroscopic infections are characterized by very low parasite densities, which are enough for a genus-level detection but may be below the sensitivity threshold of species-specific qPCR [58].

## Conclusion

Guatemala is a malaria-endemic region; however, information on malaria during pregnancy, congenital malaria, and submicroscopic malaria is scarce. This research provided insights into the burden and risk factors of submicroscopic congenital malaria, showing that submicroscopic malaria during pregnancy is a significant risk factor for submicroscopic congenital malaria. These findings highlight the importance of malaria surveillance, early diagnosis, and treatment in pregnant women, and provide a baseline for future research.

## Supplementary Information

Additional file1 (DOCX 17 kb)

## Data Availability

The data sets generated and analysed during the current study are not publicly available, as they were collected by other researchers, but are available through the corresponding author upon reasonable request.

## Abbreviations

Hb: Haemoglobin
PCR: Polymerase chain reaction
P. falciparum: *Plasmodium falciparum*
*PregVax*: *Plasmodium vivax* Infection in Pregnancy study
*P. vivax*: *Plasmodium vivax*
qPCR: Real-time polymerase chain reaction
WHO: World Health Organization
