# Supplementary material for: Prevalence of submicroscopic congenital malaria and associated risk factors in an area of low endemicity of Guatemala
Source: Malar J. 2025 Nov 27;25:9. doi: 10.1186/s12936-025-05670-6 (PMC12772006; doi:10.1186/s12936-025-05670-6)
Supplement: Supplementary file 1 — Additional file1 (DOCX 17 kb) [file 12936_2025_5670_MOESM1_ESM.docx]

SUPPLEMENTARY MATERIAL

Table Legends

Table S1. Characteristics of pregnant women from the primary study, Guatemala, 2008-2011

**Table S1.** Characteristics of the full primary cohort of pregnant women from the primary study, Guatemala, 2009-2011 (N=2009).

| **Characteristic** | **N = 2,009**^1^ |
| --- | --- |
| Age in years, median (IQR) | 23 (19, 29) |
| Number of prenatal visits when enrolled, n, (%) |  |
| 1-2 visits | 1,691 (85%) |
| >2 visits | 310 (15%) |
| Weight in pounds, (median, IQR) | 126 (116, 140) |
| Height in centimetres, (median, IQR) | 151 (147, 155) |
| Haemoglobin (g/dL) at recruitment, (median, IQR) | 11.30 (10.50, 12.00) |
| Gravidity, (median, IQR) | 2 (1, 4) |
| Gestational age in weeks at time of recruitment (median, IQR) | 27 (21, 33) |
| Trimester of pregnancy at time of recruitment (according to gestational age), n (%) |  |
| First | 131 (6.6%) |
| Second | 791 (40%) |
| Third | 1,056 (53%) |
| Method to estimate gestational age, n (%) |  |
| Date of last menstruation | 113 (5.7%) |
| Ultrasound | 241 (12%) |
| Uterine height | 1,612 (82%) |
| ^1^Median (IQR); n (%) | |

± Observations with missing data: Age (n=8), number of prenatal visits when enrolled (n=8), weight (n=3), height (n=4), haemoglobin (n=248), gravidity (n=5), gestational age (n=31), trimester of pregnancy according to gestational age (n=31) and method to estimate gestational age (n=43)
